# Papillary Thyroid Carcinoma with Lung Metastasis Arising from Dyshormonogenetic Goiter: A Case Report

**DOI:** 10.1155/2013/813167

**Published:** 2013-11-04

**Authors:** Ersin Sukru Erden, Cenk Babayigit, Ramazan Davran, Mustafa Akin, Sinem Karazincir, Nebihe Isaogullari, Mesut Demirkose, Sebahat Genc

**Affiliations:** ^1^Department of Chest Diseases, Faculty of Medicine, Mustafa Kemal University, 31000 Hatay, Turkey; ^2^Department of Radiology, Faculty of Medicine, Mustafa Kemal University, 31000 Hatay, Turkey; ^3^Department of Pathology, Faculty of Medicine, Mustafa Kemal University, 31000 Hatay, Turkey

## Abstract

Prior radiation exposure is the best known risk factor for thyroid cancers, and papillary thyroid carcinoma (PTC) may arise from dyshormonogenetic goiter. A 17-year-old female patient was admitted to the department of chest diseases with respiratory symptoms. The patient had undergone a thyroid surgery for goiter at the age of 9. A bilateral nodular opacity was detected by radiological examination. The histopathologic examination of the specimen obtained from computed tomography guided trucut biopsy was diagnosed as PTC. We present a very rare case of PTC with lung metastasis that had undergone subtotal thyroidectomy due to dyshormonogenetic goiter eight years ago.

## 1. Introduction

Thyroid cancers are the most common endocrine malign tumors. Papillary thyroid carcinoma (PTC) is the most frequent type with a ratio of 80% [1, 2]. PTC commonly metastasizes to regional lymph nodes. However, distant metastasis may rarely occur and accounts for 5% of the patients. The lungs and the bones are the most common sites for distant metastasis [3]. Major risk factors for PTC include radiation exposure, insufficient or excess dietary iodine, Cowden's disease, Gardner's syndrome, and dyshormonogenetic goiter [4–8].

Dyshormonogenetic goiter is seen in 10–20% of cases of congenital hypothyroidism. The two mechanisms are responsible for the development of dyshormonogenesis. These mechanisms are defects in synthesis and secretion of thyroglobulin and organification of iodine caused by TPO gene mutations [9]. The mechanisms are not known regarding thyroid cancer development from dyshormonogenetic goiter. However, it has been suggested that thyroid carcinoma may develop from dyshormonogenetic goiter due to long-term increased thyrotropin plasma levels [10].

In this study, we present a very rare case of PTC with lung metastasis that had undergone subtotal thyroidectomy due to dyshormonogenetic goiter eight years ago. 

## 2. Case Report

The 17-year-old nonsmoking female patient was admitted to the department of chest diseases with cough, sputum, fatigue, and chest pain. The case had undergone a subtotal thyroidectomy for goiter at the age of 9, and histopathologic examination of surgical material had revealed dyshormonogenetic goiter. Histopathologic examination revealed irregular nodular structures separated by fibrous bands; colloid containing follicles and papillary structures had nuclear hyperchromasia with the follicular epithelium, secondary follicules in the papillary structures, atherosclerotic changes in the thyroidal arteries, and hyperplastic thyroid tissue in one area. The nuclei were not in the feature of papillary carcinoma in the papillary areas. On physical examination, there was decrease in bilateral respiratory sounds.

Laboratory parameters were as follows: Hb: 12.5 g/dL, Htc: 39.0%, WBC: 10900/*μ*L, PLT: 303000/*μ*L, free T3: 4.18 (2.5–3.9) pg/mL, free T4: 0.76 (0.61–1.12) ng/dL, and TSH: 3.69 (0.34–5.60) uIU/mL. The other biochemical parameters and tumor markers were also normal. Tuberculosis bacilli were not seen in sputum examination.

**Figure 1:**
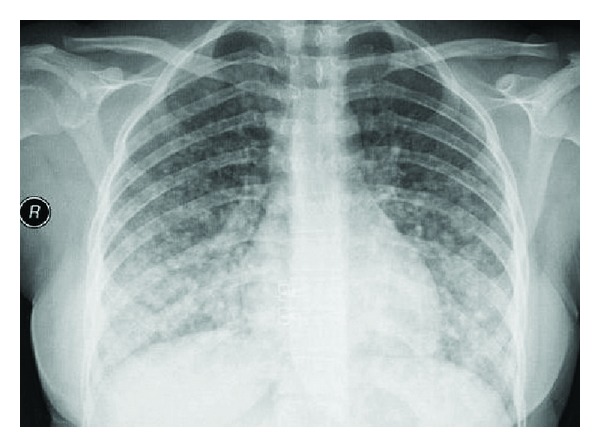
In the chest radiography, widespread nodular opacities are seen in all the zones, which are intensified in the lower zones.

**Figure 2:**
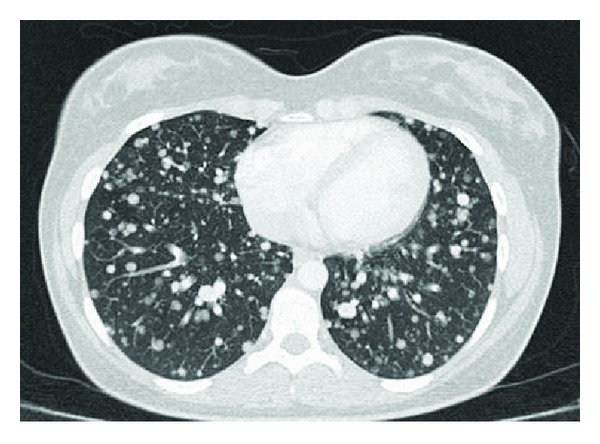
In thoracic CT, multiple atypically located nodule formations are seen in bilateral lungs.

In the chest radiography, a bilateral nodular opacity was detected (Figure 1). Thorax CT revealed multiple nodule formations that were atypically scattered along bilateral lungs (Figure 2). The neck USG displayed multiple hypoechoic and isoechoic nodules that have the greatest size of 13 × 12 mm in the thyroid gland. There were also multiple lymphadenopathies in the left cervical chain which has the greatest diameter of 13 × 11 mm.

**Figure 3:**
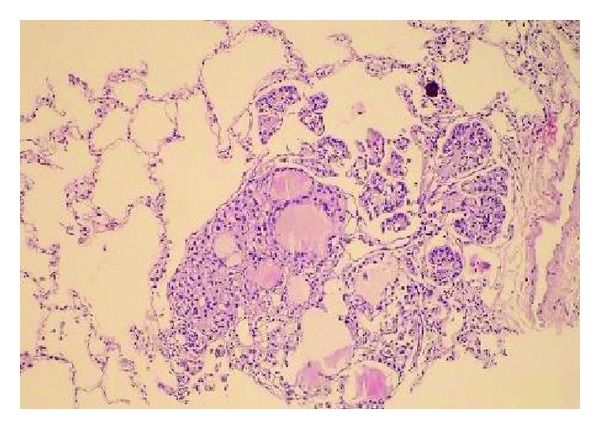
A focus of thyroid papillary carcinoma is seen while penetrating into an alveolar lumen near the arteriole. In the focus of the metastasis, the follicular and papillary structures presenting with colloid in their lumens are displayed along with the psammoma body (H&E ×100).

**Figure 4:**
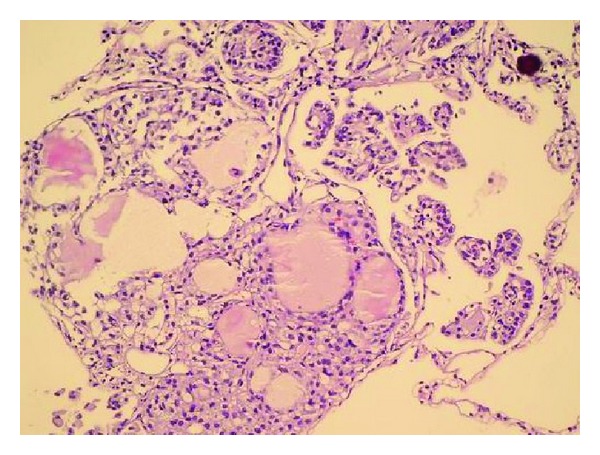
In a closer look, follicular and papillary structures, psammoma body, and intranuclear inclusions become visible as well (H&E ×200).

A diagnostic bronchoscopy was tried, but the patient could not tolerate it. The case underwent CT guided trucut biopsy on the left lung. The patient had respiratory insufficiency during followup due to pneumothorax on the left hemithorax. Therefore, she received a tube thoracostomy for pneumothorax treatment. The result of biopsy was consistent with thyroid papillary carcinoma (Figures 3 and 4). Then, she was referred to the Endocrinology and General Surgery Outpatient Clinic. Completion thyroidectomy, neck lymph node dissection, and radioactive iodine treatment were planned to the patient.

## 3. Discussion

Primary thyroid cancers are histologically divided into four groups: (I) well differentiated epithelial thyroid cancers, (II) poorly differentiated epithelial thyroid cancers, (III) medullary thyroid cancers, and (IV) rare thyroid tumors (lymphoma, sarcoma, squamous cell, etc.). Of these, well differentiated papillary and follicular cancers constitute the largest part with a rate of over 90% [11]. Also, papillary thyroid cancer is the most common type of well differentiated thyroid cancers [3]. 

PTC has a good prognosis, and it is one of the best treatable cancers, leading to a survival rate of 93% at 10 years [12]. Young patients that have smaller tumors and do not present with invasion have better prognosis [13]. Thyroid cancers are quite rare in children, and an annual incidence of 0.52/100.000 is reported for the children under the age of 19 in the USA [14]. In differentiated thyroid cancers, females have a twice higher incidence rate than males, and the mean age of diagnosis is 45 years. In the regions with insufficient iodine, follicular carcinoma is more common than papillary carcinoma. Prior radiation exposure, particularly in early childhood, is reported as a significant risk factor for papillary carcinoma. Differentiated carcinomas may be associated with Cowden's disease, Gardner's syndrome, and familial adenomatous polyposis [4]. Malignant transformation may occur in the children with congenital hypothyroidism if the risk of thyroid nodule is increased by the presence of dyshormonogenesis or iodine transport defect. Follicular carcinoma is the most common type among the cases of congenital hypothyroidism, while papillary carcinoma is very rare [5, 6, 15]. A thyroid carcinoma may be associated with a high TSH stimulation. In a study conducted with the rats, it was reported that the presence of long-time elevated TSH levels results in a thyroid carcinoma with lung metastasis [16]. In our patient, half of the thyroid gland had been removed due to the goiter at the age of 9, and 8 years later, the case was diagnosed as PTC via the histopathologic examination of CT guided biopsy due to symptoms in terms of lung metastasis.

The patient with PTC commonly presents with asymptomatic thyroid nodules. Symptoms including pain, respiratory insufficiency, stridor, vocal cord paralysis, and hemoptysis may also be seen [17]. The current patient presented with cough, sputum, fatigue, and pain in the chest. Kallel et al. revealed the case of a PTC with lymph node metastasis resulting from dyshormonogenetic goiter in a 13-year-old boy who had total thyroidectomy due to voluminous goiter associated with hypothyroidism [6]. Drut and Moreno reported the patient who was a 5-year-old girl with nongoiter congenital dyshormonogenetic hypothyroidism. The specimens obtained from the thyroid nodule which was detected in the clinical followup during the appropriate treatment were diagnosed as PTC [18]. Yashiro et al. reported a case of thyroid papillary carcinoma associated with dyshormonogenetic goiter who had been on thyroid hormone therapy since the age of three. In physical and ultrasonographic examinations, the case had a tumor in the right thyroid lobe, and PTC was detected following the total thyroidectomy [5]. In all these cases, PTC was detected via the investigations of the findings related to thyroid gland. However, in our case, PTC was detected due to the symptoms related to distant metastasis. To the best of our knowledge, this is the first case in the literature that it arose from dyshormonogenetic goiter and was diagnosed through distant metastasis.

In PTC, metastases primarily occur in regional lymph nodes. Systemic metastasis may rarely occur—most commonly in the lungs or bones—and accounts for 5% of the patients. It rarely affects the skeletal muscle, ovaries, submandibular gland, sphenoidal sinus, brain, adrenal gland, and pancreas [3]. In the present case, the metastases were firstly defined in the lungs. The lung metastases in PTC may develop as miliary or multiple or localized infiltration, widespread lymphadenopathy, or pleural effusion [19]. Our patient presented with bilateral widespread nodule formations in radiological examination.

Risk factors for distant metastases include male gender, advanced age, histologic grade, completeness of surgical resection of the primary tumor, and extrathyroidal invasion at initial examination [8]. Our patient had none of these factors; however, the completeness of surgical resection of the dyshormonogenetic goiter may be regarded as a risk factor.

Treatment of metastatic PTC cases may include the sole use of radioactive iodine therapy, surgery, thyroid hormone, radiotherapy, and chemotherapy or various combinations of these treatments [20].

In this study, we present a patient who was diagnosed with a very rare case of PTC with lung metastases arising from dyshormonogenetic goiter. It is suggested that the patients with congenital hypothyroidism and dyshormonogenetic goiter may rarely develop thyroid carcinoma and they should be closely followed up in the long term.
